# Eastern Ohio Dental Association

**Published:** 1872-04

**Authors:** D. B. Mclain

**Affiliations:** Secretary.


### ARTICLE IV.

### *Eastern Ohio Dental Association.*

BY DR. D. B. MCLAIN, Secretary.

The second semi-annual meeting of this Association was convened in Alliance, February 27th, 1872, at 1 o'clock, P. M., by the President, Dr. J. C. Whinery, of Salem, who invited the Rev. Mr. Wolf, of Alliance, to open with prayer. Members present, Whinery, Lyder, McLain, Douds, Siddal, Porter, Hole and Hisey.

The Treasurer reported having in his hands $7.80.

Dr. J. M. Porter, of Massillon, read a very able and elaborate essay on anæsthesia and respiration.

Dr. J. W. Lyder, of Alliance, read an essay on Dental Association and its benefits, pointing to the great advance made through the influence of dental associations and alluding to the damaging influence on the profession of those men who do not believe in association or who attend one meeting for pecuniary motives only, in order to class themselves as members in good standing. On motion, these views were adopted as the sentiment of the Association.

Dr. Siddal gave his method of producing anæsthesia, using chloroform and ether.

Dr. Whinery addressed the association on anæsthesia.

Dr. Lyder briefly deprecated the, in many cases, low fees for administering anæsthetics, taking into consideration the great danger always attendant.

Dr. Siddal delivered an oration on the rise and progress of the dental profession, and laudatory of the shining lights of past days.

On motion, a cordial invitation was given to members of the medical profession to participate in the discussions.

On the subject, "When is Extraction Indicated," Dr. Douds, of Canton, always extracts the second bicuspid, in case the cuspid is crowded out of the arch, provided, that it is necessary to extract and the first bicuspid is sound. As from observation he thinks the first bicuspid less subject to decay.

Dr. McLain reported cases where he had successfully treated excessive deformity of the mouth from protrusion of cuspids, the central and lateral incisors touching, by extracting the first bicuspids, preferring them on account of being able to bring the cuspids in place with less disturbance than by extracting any other tooth. If there was not room sufficient, procure it by plates or wedging, or both, using rubber ligatures in most cases to move the teeth.

Dr. Porter deprecated in strong terms the wholesale extraction of slightly diseased teeth and roots, claiming that in the maiority of cases they can be returned to health by judicious treatment.

Dr. Lyder reported a case, where a superior lateral incisor perfectly sound, closing inside the arch had received such injuries from unnatural occlusion as to cause alveolar abscess, the root being almost as black as jet when extracted.

Dr. McLain reported a case where a lady, aged 45, after the extraction of the entire upper denture, erupted a molar near the mesial, line about six line sposterior to the natural position of the right lateral, the side of the tooth presenting to the mouth, the crown in front, and a bicuspid in the natural position.

Also a case where one of three sisters had an entire denture extracted, and the other two the upper denture, all the teeth dead, not one decayed, but being a continual source of inflammation and ulceration ; many of the roots were necrosed. The first sister had lost her hearing. A depraved condition of the general was present system in all three.

Dr. Whinery reported several cases of complicated disease of maxillary sinus, treated successfully with dil. chlor. zinc and dil. carbolic acid, alternately.

On motion, the next place and time of meeting was appointed at Salem, August 29th, 1872, at 10 A. M.

A committee consisting of Drs. Coffee, Douds, and Hisey, were appointed to nominate delegates to the American Dental Convention. They selected Drs. Porter of Masillon, Whinery, of Salem, and McLain, of New Lisbon, who were thereupon elected by the Association.

The Chair appointed as essayists for the next meeting Drs. Porter and Douds. Subjects for discussion, "The Proper Treatment and Filling of Diseased Teeth and Roots," "Dental Ethics," "Specialties in Dentistry."

The following resolution by Dr. Whinery was adopted:

*Resolved*, That this Association heartily endorse the action of the State Dental Association in appointing a committee to defend the dentists of the State against the demands of the Dental Vulcanite Company under the Cumming's patent.

After thanking the Masonic Order for the use of their hall, the Association adjourned.

---

